# Expression of stem cell markers SALL4, LIN28A, and KLF4 in ameloblastoma

**DOI:** 10.1186/s13000-023-01379-9

**Published:** 2023-08-09

**Authors:** Rafaela de Albuquerque Dias, Karolyny Martins Balbinot, Maria Sueli da Silva Kataoka, Sérgio de Melo Alves Júnior, João de Jesus Viana Pinheiro

**Affiliations:** 1 Laboratory of Pathological Anatomy and Immunohistochemistry, Federal University of Pará, Belém, Pará, Brazil; 2 Cell Cultivation Laboratory, Federal University of Pará, Belém, Pará, Brazil

**Keywords:** Ameloblastoma, Neoplasm invasiveness, Stem cells, Immunohistochemistry, Immunofluorescence

## Abstract

**Background:**

Ameloblastoma (AME) is a benign odontogenic tumour of epithelial origin characterised by slow but aggressive growth, infiltration, and recurrence; it is capable of reaching large dimensions and invading adjacent structures. Stem cell research has proven to be significant in the sphere of tumour biology through these cells’ possible involvement in the aetiopathogenesis of this tumour.

**Methods:**

Immunohistochemistry was performed on AME, dentigerous cyst (DC), and dental follicle (DF) samples, and indirect immunofluorescence was performed on the AME-hTERT cell line to determine the expression of SALL4, LIN28A, and KLF4.

**Results:**

Expression of proteins related to cellular pluripotency was higher in AME cells than in DC and DF cells. The analysis revealed that the proteins in question were mainly expressed in the parenchyma of AME tissue samples and were detected in the nuclei of AME-hTERT cells.

**Conclusions:**

Stem cells may be related to the origin and progression of AME.

## Introduction

Ameloblastomas (AME) are odontogenic tumours of epithelial origin. Although classified as a form of benign tumour, AME is characterised by aggressiveness, an infiltrative nature, and a high recurrence tendency. They can grow to large dimensions and invade adjacent structures, causing significant morbidity [1–3].

According to the most recent classification by the World Health Organisation (WHO), AME can be categorised into three types: conventional, unicystic, and extraosseous/peripheral. Among these, conventional AME is the most common and most aggressive [4].

The current therapeutic options include both conservative and radical approaches [5]. Conservative treatments involve enucleation and curettage [1], which avoid relevant facial deformities [5] but have recurrence rates of up to 55% [6]. More invasive treatments involve marginal resection, which is the method of choice, considering the high recurrence rates reported for AME [1]. However, this method can result in relapse rates of up to 15% in more aggressive AME types as well as significant facial deformities [7].

Although the causes of the aggressive biological behaviour and high rate of recurrence of this benign neoplasm are not fully understood, studies involving stem cells and their possible relationship with the aetiopathogenesis of neoplasms have been relevant in elucidating this behaviour [8–10].

Stem cells can perpetuate themselves through self-renewal mechanisms and differentiate into cells in specific tissues. These mechanisms are like those that occur in tumour cells and involve similar signalling pathways. Thus, both normal stem cells and tumorigenic cells have proliferative potential and the ability to give rise to new tissues called cancer stem cells [8].

Cancer stem cells proliferate uncontrollably, driving the formation and growth of tumors, generating heterogeneous malignant cells associated with recurrence and metastasis. It is believed that cancer cells can appropriate the self-renewal machinery that is normally expressed in normal stem cells [8].

It has been reported that AME cells originate from odontogenic stem cells located in the dental lamina [11] and that tumours are likely initiated in normal stem cells that contain a perpetual minority of cancer stem cells [12, 13].

In this context, the SALL4 (Spalt-Like Transcription Factor 4), LIN28A (LIN28 homolog A), and KLF4 (Kruppel-like factor 4) proteins, which act as essential regulators of pluripotency and embryonic self-renewal and can mediate tumour progression and differentiation, are relevant biomarkers for the analysis of stem cells [14–16].

SALL4 is an essential transcription factor for the maintenance of self-renewal and pluripotency of embryonic stem cells that occurs in early embryonic development [14]. Its expression is downregulated after birth and is absent in most adult human tissues. Specifically, its expression in adult tissues is restricted to germ cells [17], except for human CD34+ haematopoietic stem cells [18]. However, its high expression and dysregulation have been demonstrated in several types of cancer [14], such as leukemia, germ cell tumours, hepatocellular carcinoma, and lung cancer [19–24], where it acts as an oncogene and participates in the processes of initiation, development, and progression of cancer [14].

LIN28A is a highly conserved ribonucleic acid (RNA)-binding protein that plays a key role in cell development and pluripotency by regulating the process of cell proliferation and differentiation [25]. It is expressed in the embryos, stem cells, and embryonic carcinoma cells [26]. Its performance occurs both physiologically (i.e. through the renewal and differentiation of stem cells, tissue repair, and glucose metabolism) and pathologically, where high levels are correlated with advanced malignant tumours, poor prognosis, and increased risk of recurrence [26–28].

KLF4 is a transcription factor that regulates cellular processes of development, differentiation, proliferation, and apoptosis. Depending on the cell type, KLF4 acts as both a tumour suppressor and an oncogene [16]. Furthermore, KLF4 is involved in stem cell renewal and maintenance of pluripotency [29, 30].

**Fig. 1:**
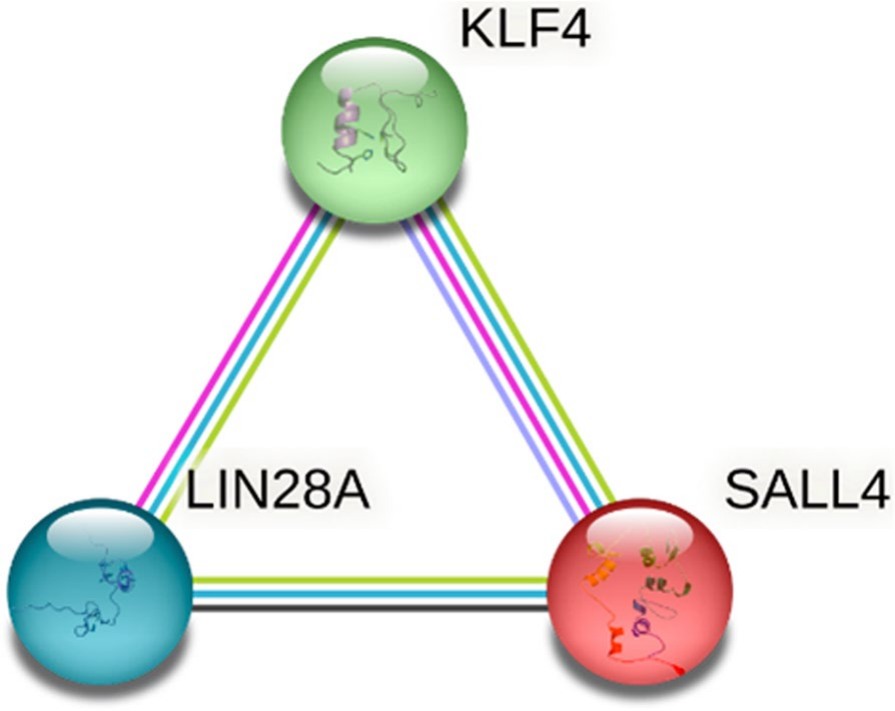
Interaction Network of SALL4, LIN28 and KLF4 proteins obtained through the STRING platform. All observed interactions between proteins (edges connecting nodes) were obtained from selected databases (light blue line) and confirmed by text mining analysis (yellow line). LIN28A and KLF4 and SALL4 and KLF4 interactions were determined experimentally (pink line). Protein homology was demonstrated between SALL4 and KLF4 (purple line), and coexpression between LIN28A and SALL4 was computationally observed from transcript–transcript interactions (black line)

The protein–protein interaction network was analysed for SALL4, LIN28A, and KLF4 proteins using bioinformatics with the STRING (Search Tool for Recurring Instances of Neighbouring Genes) platform [31]. According to the STRING platform, a direct association among them was demonstrated in all interactions obtained from the selected databases and was confirmed using text mining analysis. Interactions among LIN28A, KLF4, SALL4, and KLF4 were determined experimentally. Additionally, the platform demonstrated protein homology between SALL4 and KLF4, and computationally identified co-expression of LIN28A and SALL4 based on transcript-transcript interactions (see Fig. 1).

Understanding the molecular mechanisms underlying AME through the expression of stem cell biomarkers can help elucidate the role of these cells in the aetiopathogenesis of this tumour. Therefore, the present study aimed to evaluate the in situ and in vitro expression of stem cell markers SALL4, LIN28A, and KLF4 in AME. This is the first work to simultaneously investigate these three proteins in this benign neoplasm. 

## Methods

### Ethical aspects

This study was approved by the Comitê de Ética em Pesquisa com Seres Humanos – Universidade Federal do Pará, Belém, Pará, Brazil, Committee Reference No.: 5.490.937.

### Samples

For the in vitro study, the cell line derived from human AME, called AME-hTERT, established at the Cell Culture Laboratory of the Faculty of Dentistry, Universidade Federal do Pará (UFPA), was used [32]. For the in situ study, 21 cases of conventional AME, ten cases of dentigerous cysts (DC), and ten cases of dental follicles (DF) were collected at the Laboratory of Pathological Anatomy and Immunohistochemistry of the Graduate Program in Dentistry, Universidade Federal do Pará, and Centro Universitário do Pará (CESUPA). In the in situ study, the cases of DC and DF were used as comparative samples, considering that DC and AME are benign odontogenic lesions but present with less aggressive behaviour and a low incidence of recurrence. Meanwhile, DF is a tissue without pathological neoplastic changes of odontogenic origin.

### Cell cultivation

The cell line derived from AME, established, and characterised at the Laboratory of Cell Culture of the Graduate Program of the Universidade Federal do Pará (UFPA), was cultivated in DMEM/F-12 medium (Sigma Chemical Co., St. Louis, MO, USA), supplemented with 10% foetal bovine serum (Gibco, Carlsbad, CA, USA), 1% penicillin (Gibco®) and 0.1% antifungal (Gibco®). The cells were kept in an incubator at a temperature of 37ºC and a humid atmosphere containing 5% CO_2_. Cell proliferation was observed daily using an inverted phase-contrast microscope (Axiovert 40 C – Zeiss) equipped with a coupled camera (AxioCam MRc, Zeiss).

### Immunohistochemistry

The immunohistochemistry technique used in the present study was performed according to the following protocol. First, deparaffinization of the slides in xylene and hydration in decreasing alcohol concentrations (100%, 90%, 80% and 70%) was conducted. Then endogenous peroxidases were blocked by immersing the slides in 3% H_2_O_2_ to methanol at a 1:1 ratio for 30 min. Antigenic recovery was conducted in a Pascal pressure chamber (Dako Cytomation, Carpinteria, CA, USA) in a citrate buffer (pH 6.0) for 30 s with a temperature of 123°C and pressure of 13.5 psi. Finally, non-specific antibodies were blocked with 1% bovine serum albumin (BSA; Sigma-Aldrich) in a phosphate-buffered saline solution for 1 h.

Incubation of primary antibodies for Anti-Sall4 (1:25, mouse, Santa Cruz Biotechnology, Santa Cruz, CA, USA) was conducted for 12–14 h (overnight) and for 1 h for the Anti-LIN-28 (1:30, mouse, Santa Cruz Biotechnology ®) and Anti-GKLF (1:100, mouse, Santa Cruz Biotechnology®) in the AME, DC, and DF samples. Secondary antibodies were incubated with an Immunoprobe Plus detection system (Advanced Biosystems, San Francisco, CA, USA) for 30 min. Diaminobenzidine chromogen (ScyTek, Logan, UT, USA) was used. The slides were counterstained with haematoxylin (Sigma-Aldrich) and mounted using Permount (Fisher Scientific, Fair Lawn, NJ, USA). Testicular seminoma samples were used as positive controls. As a negative control, the primary antibody was replaced with BSA (Sigma-Aldrich) or non-immune serum.

### Immunohistochemical evaluation

The immunohistochemical (IHC) evaluation was performed by measuring the area (μm) and fraction (%) of SALL4, LIN28A, and KLF4 and labelling in the AME, DC, and DF samples. Images were obtained using an Axioscope A1 microscope (Zeiss®) equipped with an AxioCam HRC colour CCD camera (Zeiss®) with a bright field. Five areas in each sample were selected based on the quantity and morphological preservation of the parenchyma. Images were acquired using a 40x objective. Areas of the tumour parenchyma were separated and segmented using the “IHC Image Analysis Toolbox” plug in (Jie Shu, Guoping Qiu and Mohammad Ilyas, https://imagej.nih.gov/ij/plugins/ihc-toolbox/index.html) using ImageJ (public domain software developed by Wayne Rasband (NIMH, National Institutes of Health, Bethesda, MD, USA; http://rsbweb.nih.gov/ij/). After segmenting the images, the area and diaminobenzidine (DAB) staining fraction were measured, and the immunostaining differences found in AME, DC, and DF were analysed.

### Indirect immunofluorescence

AME-hTERT cells were cultured on glass coverslips in 24-well plates and subjected to indirect immunofluorescence to observe the expression of SALL4, LIN28A, and KLF4. The technique involved the following steps: cell fixation in 2% paraformaldehyde for 10 min, washing with phosphate-buffered saline (PBS), permeabilisation of the membrane with a 0.5% Triton X-100 solution (Sigma®) for 15 min, wash with PBS, incubation in PBS/1% BSA for 30 min, and incubation with primary monoclonal antibodies diluted in PBS/BSA at 1% for a minimum of 12 h and a maximum of 18 h in a humid chamber at 4ºC. The primary antibodies used were Anti-Sall4 (1:50, mouse, Santa Cruz Biotechnology®), Anti-LIN-28 (1:50, mouse, Santa Cruz Biotechnology®), and anti-GKLF (1:50, mouse, Santa Cruz Biotechnology®). To detect the primary antibody, incubation was performed in a solution containing the secondary antibody conjugated to Alexa Fluor 488 (Invitrogen, Carlsbad, CA, USA) for 1 h in a humid and dark chamber at room temperature. Nuclei were stained with Hoechst 33258 (1: 2,000, Sigma) and cytoskeletons were stained with Alexa Fluor 568 phalloidin (Life Technologies, Carlsbad, CA, USA). The coverslips were immersed in PBS and distilled water and mounted using the ProLong® Gold antifade reagent (Invitrogen®). Next, the cells were analysed using a fluorescence microscope (AxioScope.A1, Zeiss) equipped with a digital camera (AxiocamMRc, Zeiss). Images of the slides were obtained for the registration of immunoexpression using a 40x objective. As a negative control, the primary antibody was replaced with a non-immune serum.

## Statistical analysis

Data were analysed using GraphPad Prism 8 software (GraphPad Software Inc., San Diego, CA, USA). When parametric distribution was evidenced by the Shapiro-Wilk test, differences between groups were evaluated by one-way analysis of variance (ANOVA) followed by Tukey’s post-hoc test. When a non-parametric distribution was evidenced by the Shapiro-Wilk test, the differences between groups were evaluated by the Kruskal-Wallis test, followed by Dunn’s post-test of multiple comparisons. A 95% confidence interval (CI) was assumed (α = 0.05).

## Results

### Demographical and clinical data, and histopathological typing of patients with AME

In the studied sample, the mean age was 37 years. Males represented 57% of the samples. The region of greatest involvement was the mandible, totalling 95% of the cases. As for the histological types, eight cases were of the follicular type, eight of plexiform, three acanthomatous and two of granular cells (see Table 1).

**Table 1:** Demographical and clinical data, and histopathological typing of patients with AME (*n* = 21)

Case	Gender	Age	Localization	Histological type
1	Male	23	Mandible	Plexiform
2	Female	31	Mandible	Follicular
3	Male	31	Mandible	Plexiform
4	Female	27	Mandible	Plexiform
5	Male	34	Mandible	Plexiform
6	Female	32	Mandible	Follicular
7	Female	55	Mandible	Plexiform
8	Female	20	Mandible	Plexiform
9	Male	32	Mandible	Plexiform
10	Male	33	Mandible	Acanthomatous
11	Female	59	Mandible	Follicular
12	Female	47	Mandible	Acanthomatous
13	Female	14	Mandible	Follicular
14	Male	42	Mandible	Granular Cells
15	Male	43	Mandible	Granular Cells
16	Male	28	Mandible	Follicular
17	Male	27	Mandible	Acanthomatous
18	Female	62	Mandible	Follicular
19	Male	84	Upper jaw	Follicular
20	Male	-	Mandible	Follicular
21	Male	22	Mandible	Plexiform

### Immunohistochemical staining for SALL4, LIN28a, and KLF4 in ameloblastoma, dentigerous cyst, and dental follicle

**Fig. 2:**
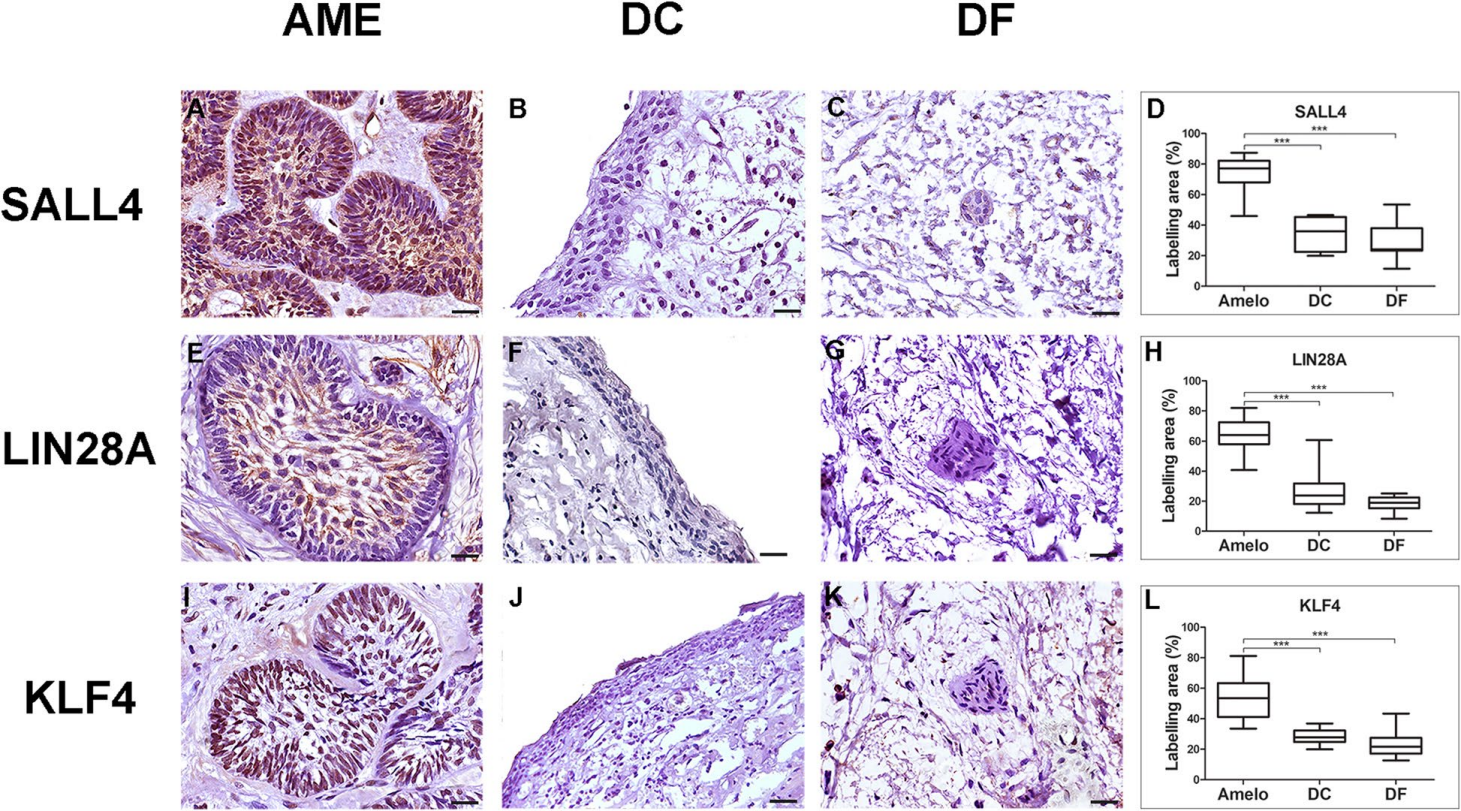
Immunohistochemical staining for SALL4, LIN28 and KLF4 in AME, DC and DF samples. Intense SALL4 immunostaining was observed in the parenchymal cells of the plexiform AME, with nuclear and cytoplasmic location (**A**). Strong LIN28A immunostaining was observed in follicular-type AME, with nuclear and cytoplasmic localization limited to the central cells of the tumor island (**E**). Intense KLF4 immunostaining in plexiform AME was observed with a nuclear location (**I**). There was a subtle labeling of the three proteins in the nucleus and cytoplasm of some epithelial cells in both DC (**B**, **F** and **J**) and DF (**C**, **G** and **K**) samples. Statistical analysis of the percentage of parenchymal marking area of the three markers (**D**, **H** and **L**) between AME, DC and DF. (***p < 0.001). Scale bar: 20 µm. AME = Ameloblastoma; DC = Dentigerous cyst; DF = Dental follicle

IHC staining for SALL4, LIN28A, and KLF4 was predominantly observed in the epithelial cells of the tumour islands (see Fig. 2). For SALL4, intense staining was observed in the tumour parenchyma in both the nucleus and cytoplasm of the cells, in the high columnar cells of the periphery, and in the central cells of the tumour island. LIN28A showed strong immunostaining with nuclear and cytoplasmic localisation limited to the central cells of the tumour island. Intense immunostaining was observed for KLF4 nuclear localisation in epithelial cells. The labelling was predominantly located in the nuclei of tall columnar cells at the periphery of the tumour island. Nuclear and cytoplasmic markings of SALL4, LIN28A, and KLF4 were also observed in some cases of DC and DF; however, the expression of SALL4, LIN28A, and KLF4 was significantly higher in AME samples compared to DC (*p* < 0.001) and DF (*p* < 0.001), as demonstrated in the statistical analysis (see Fig. 2).

### AME-hTERT lineage expressed SALL4, LIN28A, and KLF4

**Fig. 3:**
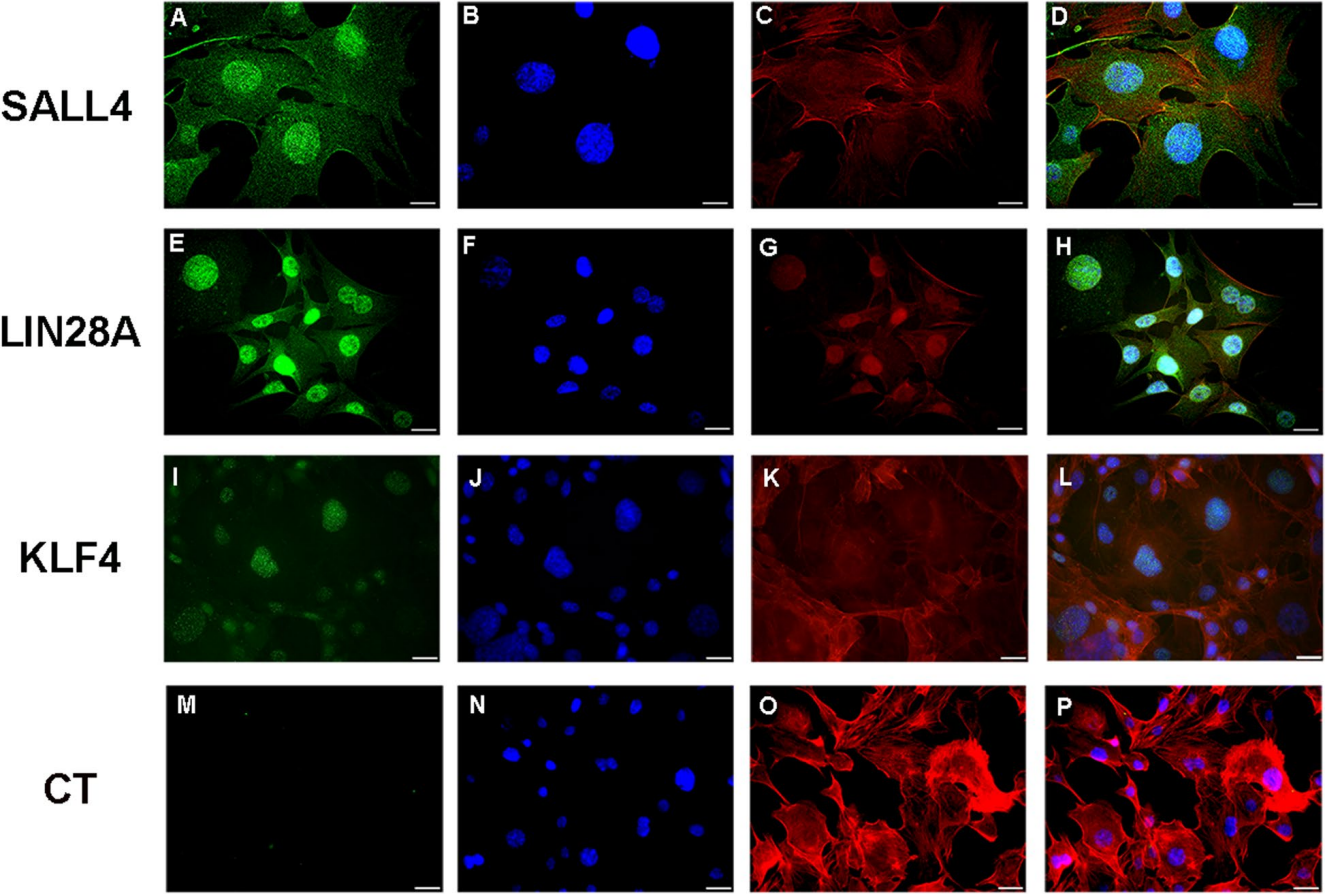
Immunofluorescence photomicrographs of SALL4, LIN28 and KLF4 in AME-hTERT strains. SALL4 showed immunoexpression with a granular pattern located in the nucleus and cytoplasm (**A**). LIN28A showed immunoexpression with a granular pattern in the nucleus and cytoplasm, with nuclear predominance (**E**). Granular expression of KLF4 was observed in the nuclei, showing faint staining in the cytoplasm (**I**). Control group (CT) (**M**). The cytoskeleton was stained with phalloidin (red), and the nuclei were stained with Hoechst 33258 (blue). Overlapping images of the expression of SALL4 (**D**), LIN28 (**H**), KLF4 (**L**) and control group (**P**). Scale bar: 20 µm

The immunofluorescence assays revealed that the AME-hTERT strain expressed SALL4, LIN28A, and KLF4 (see Fig. 3). Neoplastic cells demonstrated nuclear and cytoplasmic expression of SALL4 (see Fig. 3A), nuclear and cytoplasmic expression, with nuclear predominance of LIN28A (see Fig. 3E) and predominantly nuclear expression of KLF4 (Fig. 3I). The immunoexpression of all proteins was granular (see Fig. 3).

## Discussion

The studied proteins showed significantly higher levels of immunostaining in AME cells than in the DC and DF cells. SALL4, LIN28A, and KLF4 were expressed in the AME parenchyma, with slight staining observed in some cells of the odontogenic epithelium of DC and DF. Furthermore, immunoexpression of the studied proteins was observed in the AME-hTERT strain.

SALL4 is a transcription factor that plays a key role in maintaining pluripotency and self-renewal of embryonic stem cells [14]. It interacts with other important regulatory proteins of embryonic pluripotency — OCT4 (octamer-binding protein 4), SOX-2 (HMG-box gene 2 related to SRY), and NANOG (homeodomain protein) — forming an autoregulatory circuit in which each of these proteins regulates its own expression and that of others [33–35]. High expression of SOX-2, NANOG, and OCT4 has been demonstrated in AME [10], suggesting that these proteins may act together to maintain undifferentiated stem cells in this tumour. 

In the present study, cells from the AME tumour islands and the AME-hTERT lineage showed nuclear and cytoplasmic expression of SALL4, corroborating the marking pattern found by other studies in oral squamous cell carcinoma that suggests that this protein plays an important role in the progression of oral cancer and may serve as a potential therapeutic target [36–38]. Nuclear labelling indicated the transcriptional activity of SALL4. Such activity is associated with transcriptional repression mechanisms that prevent stem cell differentiation and increase the proliferation of undifferentiated cells [35, 39]. SALL4 cytoplasmic marking has also been demonstrated in breast cancer cells and is considered a predictor of poor prognosis [40].

Studies have shown that SALL4 protein expression is negatively regulated by miRNAs (miRNAs) belonging to the Let-7 family, particularly by miR-98, which leads to a reduction in tumour cell proliferation, indicating that miR-98 acts as a tumour suppressor that inhibits SALL4 protein expression [41, 42]. It is important to emphasise that LIN28 protein can downregulate the Let-7 microRNA family through the activation of its isoforms LIN28A or LIN28B [25, 43]. This suggests that the upregulation of LIN28 leads to the inhibition of miR-98, which, in turn, leads to the upregulation of SALL4. Therefore, the co-expression of SALL4 and LIN28A in AME observed in this study may play a significant role in tumour pathogenesis.

LIN28A is a highly conserved RNA-binding protein that plays a significant role in development, glucose metabolism, and pluripotency [44–46]. It is highly expressed in mouse embryonic stem cells, which decreases after differentiation, and in human embryonic carcinoma cells [47]. Oral squamous cell carcinoma has been demonstrated to be associated with the regulation of the proliferative and invasive activities of this neoplasm [48]. Furthermore, LIN28A has been identified as one of the four factors that convert fibroblasts into induced pluripotent stem cells, corroborating the role of this protein in pluripotent stem cells [49].

In this study, LIN28A immunostaining showed nuclear and cytoplasmic localisation limited to the central cells of the AME tumour islands. The same expression pattern was observed for the AME-hTERT strain. LIN28A is predominantly cytoplasmic and located on ribosomes, P bodies, and cytoplasmic stress granules [50]. The cytoplasmic expression found in the present study may be associated with its performance in the recruitment of terminal uridylyl transferase (TUTase4) ZCCHC11, which inhibits Let-7 processing in the cytoplasm and selectively blocks the expression of Let-7 miRNAs and their functions tumour suppressors, acting as an oncogene and promoting tumorigenesis [25, 51]. This action has been demonstrated in embryonic stem cells, suggesting a significant role of LIN28A in inhibiting cell differentiation through miRNAs in stem cells and certain types of cancer [52].

Nuclear expression of LIN28A was observed when both RNA-binding domains were mutated [50]. A model has been proposed in which LIN28A regulates the post-transcriptional processing of its mRNA targets by first binding to these targets in the nucleus and subsequently transporting them between ribosomes, P bodies, or stress granules for translation regulation, depending on the environmental conditions [50, 53]; however, more studies are needed to better understand this process.

The central region of the AME tumour islands, which exhibits greater LIN28A labelling, is more prone to hypoxia. As the tumour progresses, the concentration of oxygen in the microenvironment around the tumour cells decreases, leading to intratumoural hypoxia [54]. In response to this condition, hypoxia-induced factor-1 alpha (HIF-1α) regulates the expression of genes that help cells adapt to this environment [55]. Studies have indicated that HIF-1α is overexpressed in AME, suggesting that hypoxia is related to proliferation and invasion of the solid areas of this tumour [56–58]. It has been shown that HIF-1α binds directly to the LIN28A promoter and induces its transcription [59] and that hypoxia is capable of inducing the expression of stem cell markers in cancer cell lines, thereby contributing to the dedifferentiation and reprogramming process that induces the formation of cancer stem cells [59–61]. From this, we can infer that the expression of LIN28A in the central cells of the AME tumour island close to the high columnar cells in the periphery may be associated with the adaptive response of tumour cells to hypoxia, inducing the dedifferentiation of peripheral cells, and thus promoting greater proliferation and invasion.

KLF4 is an essential transcription factor in the regulation of cellular processes (e.g. development, differentiation, proliferation, and apoptosis) [16] and in the renewal of stem cells and maintenance of pluripotency [29, 30]. It has been used as a reprogramming factor for fibroblasts and odontoblasts in induced pluripotent stem cells along with LIN28A [62, 63]. In different cell types, KLF4 functions as both a tumour suppressor and an oncogene [16]. Increased expression has been reported in human head and neck squamous cell carcinoma and is associated with poor prognosis and aggressiveness, corroborating its oncogenic role [64, 65]. In contrast, Land et al. [66] found an association between high KLF4 expression and a favourable prognosis. Another study reported the role of KLF4 in oral squamous cell carcinoma, in which mechanisms of action were described as both tumour suppressors and oncogenes [67]. Some scholars believe that the function of KLF4 as an oncogene or tumour suppressor is modulated by its complex interactions with several tumour microenvironments [68].

In the present study, intense nuclear immunostaining for KLF4 was observed in AME, predominantly in the tall columnar cells located on the periphery of the tumour island. In the AME-hTERT strain, nuclear expression of KLF4 with mild cytoplasmic expression was observed. KLF4 is mainly located in the nucleus, but its cytoplasmic localisation has also been reported in prostate and oral cancers [65, 68, 69]. Increased KLF4 nuclear expression has been associated with poor prognosis in patients with breast and head and neck cancer [64, 70]. However, another study suggested that the downregulation of KLF4 is associated with the progression of oral carcinoma [71]. Considering the ambiguity of this transcription factor, further studies are required to assess the role of KLF4 in AME.

The findings of the present study indicate that SALL4 and LIN28A may play a significant role in the biological behaviour of AME, suggesting a possible role for stem cells in the genesis and progression of AME. The KLF4 transcription factor plays a context-dependent role in carcinogenesis and may be up or downregulated in distinct types of cancer. Therefore, its role in AME needs to be better understood. However, considering its expression together with that of other studied proteins, we suggest its participation and interaction as an oncogene. Although these results are promising, mechanistic and in vivo studies are required to confirm these hypotheses and elucidate the underlying molecular mechanisms. Understanding these mechanisms may have significant implications for the diagnosis, prognosis, and treatment of AME, thus opening up new possibilities for personalised and effective therapies.

## Conclusions

To the best of our knowledge, this is the first study to evaluate the expression of SALL4, LIN28A, and KLF4 proteins in a benign odontogenic tumour. The study results verify the expression of these stem cell markers in AME neoplastic cells by IHC and in the AME-hTERT cell line by immunofluorescence, suggesting the possible participation of stem cells in the origin, progression, and recurrence of this tumour.

## Data Availability

The datasets used and/or analysed in the current study are available from the corresponding author upon reasonable request.

## Abbreviations

AME: Ameloblastoma
AME-hTERT: Cell line derived from human AME
ANOVA: Analysis of variance
BSA: Bovine serum albumin
CI: Confidence interval
DAB: Diaminobenzidine
DC: Dentigerous cysts
DF: Dental follicles
HIF-1 α: Hypoxia-induced factor-1 alpha
IHC: Immunohistochemistry
KLF4: Kruppel-like factor 4
LIN28A: LIN28 homolog A
miRNAs: MicroRNAs
NANOG: Homeodomain protein
OCT4: Octamer-binding protein 4
PBS: Phosphate-buffered saline
RNA: Ribonucleic acid
SALL4: Spalt-like transcription factor 4
SOX-2: HMG-box gene 2 related to SRY
TUTase4: Terminal uridylyl transferase
UFPA: Universidade Federal do Pará
WHO: World Health Organisation
